# Case Report: Three pathogenic molecular findings in a patient with myotonia congenita, pseudohypoparathyroidism, and a glaucoma-suspect phenotype

**DOI:** 10.3389/fendo.2026.1850762

**Published:** 2026-08-14

**Authors:** Noha N. Mukhtar, Deema Alturki, Allianah Benito, Balgees Alghamdi, Ali Alshehri, Ali S. Alzahrani

**Affiliations:** 1 Department of Medicine, King Faisal Specialist Hospital & Research Centre, Riyadh, Saudi Arabia; 2 Molecular Endocrine Section, Research and Innovation, King Faisal Specialist Hospital & Research Centre, Riyadh, Saudi Arabia; 3 Department of Neuroscience, King Faisal Specialist Hospital & Research Centre, Riyadh, Saudi Arabia

**Keywords:** Albright hereditary osteodystrophy, *CLCN1*, *CYP1B1*, glaucoma suspect, *GNAS*, hypocalcemia, myotonia congenita, pseudohypoparathyroidism

## Abstract

**Background:**

The presence of multiple rare Mendelian disorders in a single patient may mask clinical recognition when phenotypes overlap. We describe a patient with longstanding myotonia congenita due to a *CLCN1* variant in whom an incidental discovery of severe hypocalcemia led to the diagnosis of *GNAS*-related pseudohypoparathyroidism (PHP). Exome reanalysis also identified an incidental homozygous pathogenic *CYP1B1* variant associated with autosomal recessive glaucoma.

**Case presentation:**

A 23-year-old man born to consanguineous parents was referred for management of chronic myotonia congenita, manifested by delayed motor milestones, frequent falls, contractures, and muscle hypertrophy. Electrophysiological assessment demonstrated myotonic discharges, and whole-exome sequencing (WES) identified a homozygous *CLCN1* splice-site variant (NM_000083.3:c.1167-10T>C) known to cause myotonia congenita. During subsequent evaluation, he was found to have severe hypocalcemia, hyperphosphatemia, markedly elevated parathyroid hormone, basal ganglia and dentate calcifications, brachydactyly, subcutaneous calcifications, and mild hypothyroidism, raising suspicion for PHP.

**Genetic and ophthalmologic findings:**

Reanalysis of WES data demonstrated a heterozygous frameshift *GNAS* variant (NM_080425.4:c.2494_2497delCTGA) and a homozygous *CYP1B1* variant (NM_000104.4:c.182G>A; p.Gly61Glu) in addition to the *CLCN1* defect. Sanger sequencing confirmed all three variants. The *CYP1B1* finding prompted glaucoma-specialist evaluation. Visual acuity was 20/22 in the right eye and 20/25 in the left eye; intraocular pressures were 8 and 12 mmHg, respectively. The corneas were clear, cup-to-disc ratios were 0.4 and 0.5, and average retinal nerve fiber layer thicknesses were 74 and 75 µm. No definite glaucomatous damage was identified, and the patient was classified as a glaucoma suspect. After treatment with calcium, calcitriol, and ergocalciferol, serum calcium improved, parathyroid hormone levels declined, creatine kinase normalized, and the patient reported improved muscle stiffness and function.

**Conclusion:**

This case demonstrates two clinically expressed Mendelian disorders together with a third actionable molecular finding. It highlights the importance of careful phenotyping, molecular testing, and cautious genotype-phenotype interpretation, particularly in consanguineous populations.

## Introduction

Nondystrophic myotonias are rare skeletal muscle channelopathies caused mainly by pathogenic variants in *SCN4A* or *CLCN1* and characterized clinically by delayed muscle relaxation after voluntary contraction, resulting in muscle stiffness, pain, fatigue, and episodic weakness (1, 2). *CLCN1*-associated-myotonia congenita is a specific nondystrophic myotonia caused by reduced sarcolemmal chloride conductance due to pathogenic *CLCN1* variants and may be inherited as either an autosomal dominant or an autosomal recessive disorder (3).

Pseudohypoparathyroidism (PHP) is a rare disorder of parathyroid hormone (PTH) signaling, most commonly associated with defects at the *GNAS* locus (4). It is characterized biochemically by hypocalcemia, hyperphosphatemia, and elevated circulating PTH, and clinically by variable features of Albright hereditary osteodystrophy (AHO) such as brachydactyly, short stature, subcutaneous ossification or calcification, and neurocognitive impairment (4–7). Resistance to other hormones, particularly thyroid-stimulating hormone (thyrotropin), may also occur (7). These disorders fall within the spectrum of inactivating PTH/PTHrP signaling disorders (iPPSDs) (6, 7).

Primary congenital glaucoma is a developmental glaucoma that usually presents in infancy or early childhood with elevated intraocular pressure, corneal enlargement or clouding, and progressive optic neuropathy. Biallelic pathogenic variants in *CYP1B1* are among its most common genetic causes. However, *CYP1B1*-associated disease shows variable age at onset and expression of the disease, and the same biallelic genotype may be associated with congenital glaucoma, juvenile open-angle glaucoma, late-onset glaucoma, or a glaucoma-suspect phenotype. Accordingly, a pathogenic genotype should be interpreted together with the ophthalmologic phenotype (8, 9).

The co-occurrence of multiple unrelated pathogenic molecular findings in a single patient is exceptional. We report a patient in whom longstanding myotonia congenita obscured recognition of severe hypocalcemia due to PHP. Genomic reanalysis established the molecular basis of both disorders and additionally identified a homozygous pathogenic *CYP1B1* variant that prompted ophthalmologic surveillance, although a definitive clinical diagnosis of congenital or open-angle glaucoma was not established, a diagnosis of glaucoma suspect was rendered.

## Case presentation

A 23-year-old male was referred to a neurology clinic in January 2024 for a second opinion regarding longstanding myotonia. He was born at term by cesarean section after an uncomplicated pregnancy. Symptoms began gradually in infancy, with delayed motor milestones, frequent falls, and delayed independent walking at 3 years of age. He reported no dysphagia, dysarthria, or respiratory symptoms. He had poor school performance. His parents were first-degree cousins. According to the family history, a brother and a sister had similar longstanding muscular symptoms consistent with myotonia congenita; however, the siblings were not available for formal neurological examination.

Neurological examination showed generalized myotonia with contractures involving both hands, wrists, and feet. In the upper limbs, muscle power was reduced to 4/5 with absent deep tendon reflexes and mild hypertonia. Lower-limb power was preserved, but there was marked bilateral muscular hypertrophy. Cranial nerve and sensory examinations were normal.

**Figure 1:**
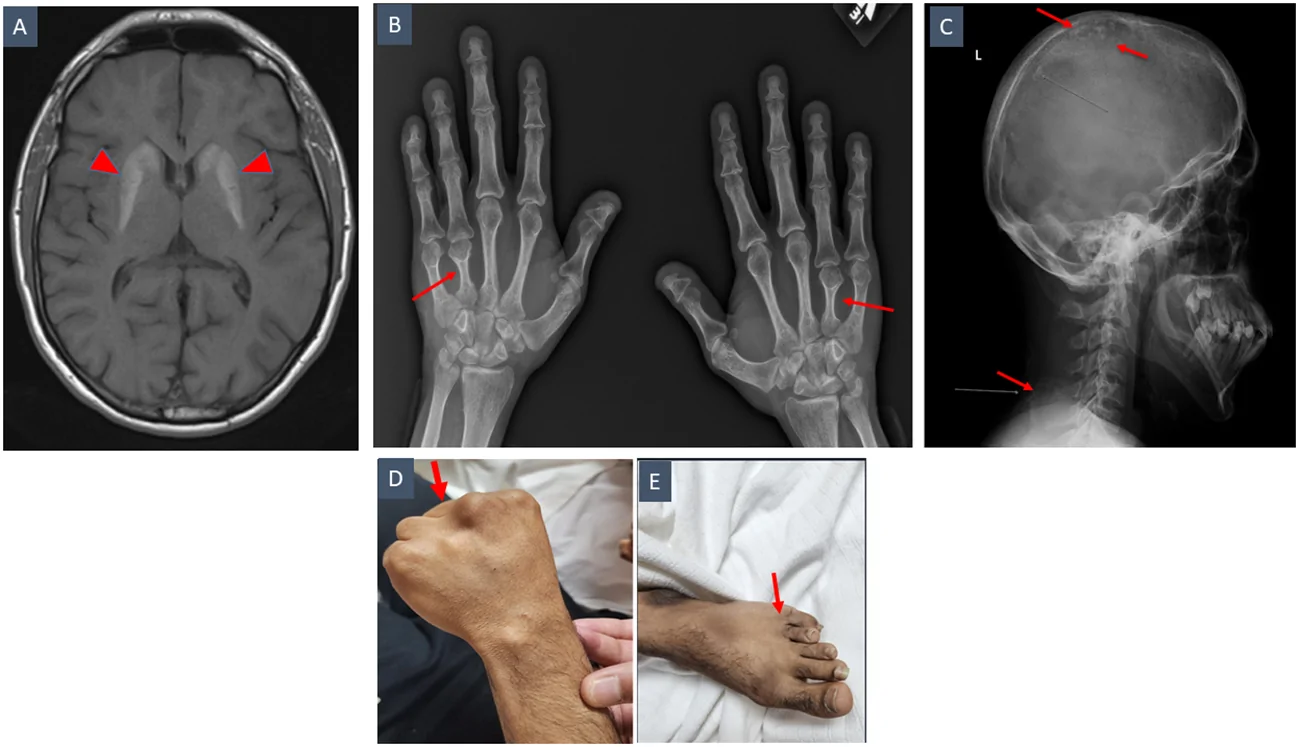
Axial T1-weighted brain MRI demonstrating symmetric hyperintense signal (arrowheads) in the bilateral basal ganglia **(A)**; hand radiographs showing bilateral shortening of the fourth and fifth metacarpal bones (arrows) **(B)**; lateral skull/neck radiograph showing intracranial and cervical soft-tissue calcifications (arrows) **(C)**; clenched left fist demonstrating the characteristic knuckle dimpling caused by shortening of the fourth and fifth metacarpal bones (arrows) **(D)**; and photograph of the left foot demonstrating shortening of the fourth and fifth metatarsal bones (arrows) **(E)**.

Initial laboratory testing showed normal blood counts and renal function, but creatine kinase (CK) was approximately four times the upper limit of normal (799 U/L; reference range 24–195 U/L). Nerve conduction and electrophysiologic studies demonstrated myotonic discharges, supporting the clinical suspicion of a nondystrophic myotonia. Whole-body MRI showed preserved muscle bulk in the upper and lower limbs. Brain MRI, however, revealed bilateral symmetric calcifications involving the basal ganglia, thalami, dentate nuclei, and cerebellar white matter, suggesting an underlying metabolic disorder (**Figure 1**). Although the distribution and extent of intracranial calcifications suggested an underlying metabolic disorder, the initial diagnostic focus remained on the longstanding neuromuscular disorder because the clinical history, examination, electrophysiologic myotonic discharges, family history, and subsequent identification of a homozygous *CLCN1* variant strongly supported myotonia congenita. Serum calcium, phosphate and PTH abnormalities were not initially considered until severe hypocalcemia was identified during subsequent cardiac assessment. This sequence likely reflected diagnostic overlook and fragmentation of evaluations across specialties.

Cardiac evaluation showed a normal sinus rhythm with left anterior fascicular block. Echocardiography demonstrated an aneurysmal atrial septum with a small secundum atrial septal defect and left-to-right shunt, findings that were confirmed by cardiac MRI without evidence of a hemodynamically significant intracardiac shunt.

Whole-exome sequencing (WES) initially identified a homozygous *CLCN1* splice-site variant (NM_000083.3:c.1167-10T>C). Based on the clinical, electrophysiological, and molecular evaluation, the patient was diagnosed with myotonia congenita. He was treated with lamotrigine initially and shifted to mexiletine at a later time with minimal improvement.

During subsequent cardiology assessment, he was incidentally found to have severe hypocalcemia (serum calcium 1.21 mmol/L; reference range 2.1-2.6 mmol/L) in the setting of an elevated plasma albumin of 49.5 g/L (reference range, 35-45) and preserved renal function. PTH was markedly elevated at 494 ng/L (reference range 15–65 ng/L), phosphate was elevated at 2.22 mmol/L (reference range 0.8-1.4 mmol/L), and 25-hydroxyvitamin D was low at 23 nmol/L (reference range 30–120 nmol/L). Mild hypothyroidism was also present (TSH 8.4 mU/L, reference range 0.2-4.2; free T4 10.4 pmol/L, reference range 12-22), consistent with partial thyrotropin resistance. The hypothalamic-pituitary-gonadal axis was normal. He was referred to endocrinology for assessment and treatment.

**Figure 2:**
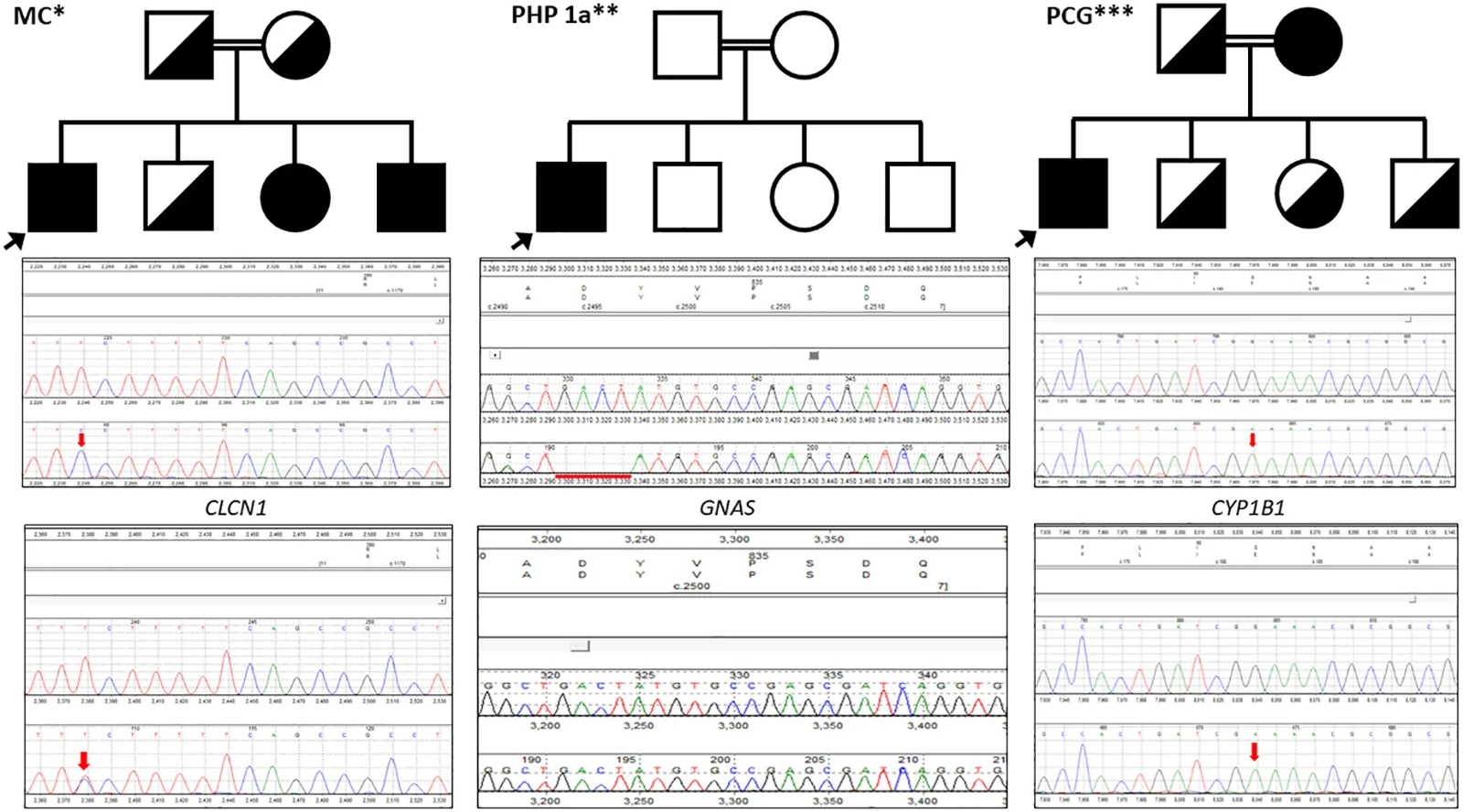
Pedigree and Sanger sequencing findings in the proband and his mother. The upper panel shows the family pedigree and segregation of the *CLCN1*, *GNAS*, and *CYP1B1* variants. The middle and lower panels show Sanger sequencing chromatograms from the proband and mother, respectively. Reference sequences are shown above patient sequences, with variants indicated by arrows or lines. Identified variants include the *CLCN1* splice-site variant NM_000083.3:c.1167-10T>C (homozygous in the proband and heterozygous in the mother), the *GNAS* deletion NM_080425.4:c.2494_2497delCTGA (present only in the proband), and the *CYP1B1* missense variant NM_000104.4:c.182G>A (homozygous in both). MC, myotonia congenita; PHP1a, pseudohypoparathyroidism type 1a; *CYP1B1*, cytochrome P450 family 1 subfamily B member 1.

On endocrine evaluation, the patient reported longstanding muscle spasms but no seizures or paresthesia. His height was 149 cm, weight 57 kg, and body mass index 25.7 kg/m². He had a short, broad neck, bilateral brachydactyly with shortening of the fourth and fifth fingers and toes (**Figure 1**), generalized muscle stiffness and contractures, and palpable hard subcutaneous nodules over the posterior cervical region and anterior shins. Chvostek sign was positive, whereas Trousseau sign was absent. His parents were first-degree cousins, and a brother and a sister had similar muscular symptoms consistent with myotonia congenita (**Figure 2**). None of his siblings had similar somatic features, including brachydactyly or subcutaneous nodules.

The combination of severe hypocalcemia, hyperphosphatemia, elevated PTH, intracranial calcification, brachydactyly, subcutaneous calcifications, and mild hypothyroidism raised a strong suspicion for AHO with associated PTH resistance (PHP). The patient was initially treated with intravenous calcium, followed by oral calcium carbonate. Vitamin D deficiency was treated with calcitriol 0.5 μg twice daily and oral ergocalciferol 50,000 IU weekly. Corrected calcium improved into the near-normal range (1.9-2.09 mmol/L), PTH levels declined in parallel with calcium correction, and CK normalized from 799 to 169 U/L. The patient also reported substantial improvement in muscle spasms and day-to-day functionality. The incidental homozygous *CYP1B1* finding prompted referral to a glaucoma specialist. No history of infantile epiphora, photophobia, corneal clouding, ocular enlargement, glaucoma surgery, or major visual impairment was elicited. Visual acuity was 20/22 in the right eye and 20/25 in the left eye. Intraocular pressures were 8 mmHg in the right eye and 12 mmHg in the left eye. Slit-lamp examination was unremarkable bilaterally, with clear corneas and lenses and deep, quiet anterior chambers. Fundoscopic examination demonstrated clear vitreous and attached retinas. The cup-to-disc ratio was 0.4 in the right eye and 0.5 in the left eye. Central corneal thickness measured 571 µm and 586 µm, and average retinal nerve fiber layer thicknesses were 74 µm and 75 µm in the right and left eyes, respectively. Gonioscopy, corneal-diameter measurements, and formal visual-field testing were not available. The findings did not establish primary congenital glaucoma or primary open-angle glaucoma; the patient was classified as a glaucoma suspect and is undergoing long-term surveillance (**Table 1**).

**Table 1:** Summary of diagnostic timeline. The major diagnostic events are summarized below:

Stage	Key findings and diagnostic consequence
Infancy to childhood	Delayed motor milestones, frequent falls, delayed independent walking at 3 years, and persistent muscle stiffness and contractures.
Neurological evaluation at 23 years	Generalized myotonia, CK 799 U/L, and electrophysiologic myotonic discharges supported nondystrophic myotonia.
Initial molecular diagnosis	WES identified homozygous *CLCN1* c.1167-10T>C, confirming myotonia congenita.
Cardiology assessment	Severe hypocalcemia was discovered incidentally, prompting endocrine referral.
Endocrine reassessment	Hypocalcemia, hyperphosphatemia, markedly elevated PTH, mild TSH resistance, intracranial calcifications, brachydactyly, and subcutaneous calcifications supported PHP1a/AHO.
Exome reanalysis and segregation	A heterozygous pathogenic *GNAS* frameshift and homozygous pathogenic *CYP1B1* p.Gly61Glu were identified and confirmed by Sanger sequencing.
Ophthalmology assessment	Normal intraocular pressure and no definitive glaucomatous damage; classified as a glaucoma suspect and scheduled for longitudinal surveillance.
Treatment response	Calcium, calcitriol, and ergocalciferol improved calcium levels, reduced PTH, normalized CK, and improved muscle symptoms.

## Genetic analysis

Reanalysis of WES confirmed the clinical diagnosis of PHP type 1a and demonstrated a coexisting heterozygous *GNAS* deletion with frameshift and truncation (NM_080425.4:c.2494_2497delCTGA; p.Asp832MetfsTer14). In addition, it showed the homozygous *CLCN1* splice-site variant (NM_000083.3:c.1167-10T>C) and a third homozygous *CYP1B1* variant (NM_000104.4:c.182G>A; p.Gly61Glu). The *CYP1B1* result represents a biallelic disease-associated genotype within the *CYP1B1*-related glaucoma spectrum but, in the absence of a diagnostic ophthalmologic phenotype, does not by itself establish primary congenital glaucoma. Family screening showed that the mother was heterozygous for the *CLCN1* variant, homozygous for the *CYP1B1* variant, and negative for the *GNAS* variant. She had no known glaucoma diagnosis by history, but a formal ophthalmologic examination was not available; therefore, she was not classified as clinically unaffected. The father was heterozygous for *CLCN1* and *CYP1B1* and negative for the *GNAS* variant. One brother was heterozygous for both *CLCN1* and *CYP1B1* and negative for the *GNAS* variant. Another brother and a sister were heterozygous for *CYP1B1*, homozygous for *CLCN1*, and negative for the *GNAS* variant. All variants were confirmed by Sanger sequencing (**Figure 2**). Applying the ACMG/AMP criteria, the *CLCN1* variant NM_000083.3:c.1167-10T>C was classified as pathogenic/likely pathogenic (PM3 Strong, PM2 Supporting, PP3, PP4, PP1 Supporting), supported by prior reports in affected individuals, rarity in population databases, a highly concordant phenotype with longstanding myotonia, elevated CK and EMG myotonic discharges, and family segregation consistent with recessive inheritance.

The *GNAS* deletion NM_080425.4:c.2494_2497delCTGA was classified as pathogenic (PVS1, PM2 Supporting, PM6/PS2, PP4). It causes a frameshift and premature termination, consistent with loss of function, a known mechanism of *GNAS*-related disease. The variant is rare, previously reported, absent in tested relatives, and the phenotype was highly specific for PHP1a/AHO.

The *CYP1B1* variant NM_000104.4:c.182G>A (p.Gly61Glu) was classified as pathogenic using ACMG/AMP evidence including PM3 Very Strong, PM1, PP3, and PS3 Supporting. This classification is supported by its repeated observation in homozygous or compound heterozygous individuals with *CYP1B1*-related glaucoma, its location in a functionally important hinge region, concordant computational predictions, and functional evidence of reduced protein activity. Importantly, classification of the variant as pathogenic describes the allele and does not, by itself, establish a specific glaucoma phenotype in the proband.

## Discussion

This young man has two clinically established rare genetic disorders—myotonia congenita and PHP type 1a—together with an incidental biallelic pathogenic *CYP1B1* finding that confers a clinically well known risk of glaucoma. This combination is unusual and reflects the high burden of recessive disease in highly consanguineous populations (10–12). Myotonia congenita is a disorder characterized by hyperexcitability of the skeletal muscle membrane (3). Under normal conditions, skeletal muscle fibers have a high resting chloride conductance that counterbalances potassium conductance and stabilizes membrane excitability. In patients with myotonia congenita, mutations in the voltage-gated chloride channel reduce chloride conductance, allowing potassium to accumulate in the transverse tubules of the sarcolemma. This promotes repetitive depolarization, leading to delayed muscle relaxation and prolonged contraction (1, 2). The current patient has a splice-site variant in *CLCN1* intron 10, 10 nucleotides upstream of exon 11. Although noncanonical, this variant has been reported in several patients with myotonia congenita (13–15), is listed as pathogenic in HGMD and ClinVar (16, 17), and is extremely rare in gnomAD.

The term PHP comprises a heterogeneous group of rare disorders caused by impaired PTH signaling, most commonly due to genetic or epigenetic abnormalities affecting the *GNAS*/Gsα pathway (7). Clinically, PHP is characterized by resistance to PTH, leading to hypocalcemia, hyperphosphatemia, and elevated circulating PTH concentrations, often in association with features of AHO such as brachydactyly, short stature, obesity, and ectopic ossifications (4, 6, 7). Additional endocrine abnormalities, particularly TSH resistance, are frequent (7). Because the phenotype is variable and may evolve over time, diagnosis relies on careful matching of biochemical findings with characteristic clinical features, followed by molecular confirmation when available. The molecular basis most commonly involves either inactivating *GNAS* variants or imprinting defects at the *GNAS* locus, including methylation abnormalities and structural defects involving nearby regulatory regions such as STX16 (4, 6, 7). Owing to the complex parent-of-origin imprinting of *GNAS*, the phenotype depends on whether the defective allele is maternally or paternally inherited; maternal defects are typically associated with PTH resistance and classic features of PHP, whereas paternal defects are more often associated with AHO without significant hormone resistance (4, 6). In our patient, the *GNAS* alteration was a 4-base-pair deletion leading to a frameshift and premature termination 14 codons downstream (p.Asp832MetfsTer14). This variant has been described in patients with variable phenotypic expression, including PHP type 1a, progressive osseous heteroplasia, and osteoma cutis with pseudopseudohypoparathyroidism (18–20). This is consistent with our patient’s classic PHP type 1a phenotype. The variant was absent in both parents and all tested siblings, supporting a *de novo* event. Although parent-of-origin was not directly tested, the patient’s severe PTH resistance, partial TSH resistance, and classic AHO features suggest that the *de novo* variant most likely affected the maternally derived *GNAS* allele.

Biallelic pathogenic *CYP1B1* variants most commonly cause primary congenital glaucoma, and p.Gly61Glu is a recurrent disease-associated allele in Saudi Arabia and neighboring populations (8, 9). Nevertheless, *CYP1B1*-associated glaucoma shows variable expressivity. Studies of families carrying biallelic disease-associated genotypes have identified individuals with classic congenital glaucoma, juvenile or adult open-angle glaucoma, glaucoma-suspect findings, and, less commonly, no detectable disease at the time of examination (21, 22). The homozygous p.Gly61Glu variant has specifically been reported in Saudi patients with juvenile-onset open-angle glaucoma, demonstrating that this allele is not restricted to an infantile phenotype (23). Conversely, a large Saudi series suggested that most established pathogenic *CYP1B1* alleles are highly penetrant overall, supporting continued surveillance even when early examination is nondiagnostic (24). In the present patient, no typical infantile history was elicited, the corneas were clear, intraocular pressures were low-normal, and there was no definite glaucomatous optic neuropathy. These findings do not support a clinical diagnosis of primary congenital glaucoma. Primary or juvenile open-angle glaucoma also remains unconfirmed and no definite structural progression has been demonstrated. We therefore classify the patient as having a biallelic pathogenic *CYP1B1* genotype with a “glaucoma-suspect” phenotype, rather than confirmed congenital or open-angle glaucoma. The mother, who carries the same homozygous variant, cannot be considered unaffected without formal ophthalmologic assessment. Both individuals will undergo periodic specialist surveillance, including intraocular-pressure measurement and ophthalmologic assessment.

In addition to the unusual co-occurrence of two rare genetic disorders and a third pathogenic molecular finding in a single patient, this case illustrates how one well-established rare diagnosis can delay recognition of a second clinically important disorder when symptoms overlap. The patient was referred because of longstanding myotonia and was found to harbor a causative homozygous *CLCN1* variant. However, severe hypocalcemia, hyperphosphatemia, markedly elevated PTH, brachydactyly, subcutaneous calcifications, and intracranial calcifications were not explained solely by myotonia congenita. Careful clinical evaluation and reinterpretation of the phenotype and its related genotype were therefore key to identifying coexisting PTH resistance (7).

The clinical course also suggests that hypocalcemia contributed considerably to the severity of his neuromuscular symptoms. Hypocalcemia can cause muscle cramps, weakness, paresthesias, tetany, and, in some cases, CK elevation due to hypocalcemia-associated myopathy or rhabdomyolysis. In our patient, the improvement in neuromuscular symptoms and normalization of CK after calcium correction support a substantial reversible metabolic component. Therefore, the acute neuromuscular phenotype should not be attributed solely to the CLCN1-related disorder-related disorder. However, the persistent biochemical pattern of PTH resistance, partial TSH resistance, and classic AHO features remained consistent with PHP type 1a.

## Conclusion

In summary, we describe a patient with two clinically confirmed rare genetic disorders—*CLCN1*-related myotonia congenita and *GNAS*-related PHP type 1a—and a third important molecular finding, a homozygous pathogenic *CYP1B1* variant associated with glaucoma susceptibility. The case underscores several important lessons: coexisting endocrine and neuromuscular disorders may mask one another; multiple pathogenic molecular findings may occur in consanguineous populations; genomic results must be interpreted in the context of the clinical phenotype; and incidental findings may justify surveillance even when diagnostic criteria for disease are not yet met. Careful phenotyping and periodic reassessment remain essential when the molecular and clinical findings are not fully concordant.

## Data Availability

The data supporting the findings of this case report are available from the corresponding author on reasonable request, subject to institutional and ethical restrictions.
